# Increased Monocyte-Derived CD11b^+^ Macrophage Subpopulations Following Cigarette Smoke Exposure Are Associated With Impaired Bleomycin-Induced Tissue Remodelling

**DOI:** 10.3389/fimmu.2021.740330

**Published:** 2021-09-16

**Authors:** Steven P. Cass, Olivia Mekhael, Danya Thayaparan, Joshua J. C. McGrath, Spencer D. Revill, Matthew F. Fantauzzi, Peiyao Wang, Amir Reihani, Aaron I. Hayat, Christopher S. Stevenson, Anna Dvorkin-Gheva, Fernando M. Botelho, Martin R. Stämpfli, Kjetil Ask

**Affiliations:** ^1^Medical Sciences Graduate Program, McMaster University, Hamilton, ON, Canada; ^2^Department of Medicine, Firestone Institute for Respiratory Health, McMaster University and The Research Institute of St. Joe’s Hamilton, Hamilton, ON, Canada; ^3^Department Biochemistry and Biomedical Sciences, McMaster University, Hamilton, ON, Canada; ^4^Janssen Disease Interception Accelerator, Janssen Pharmaceutical Companies of Johnson and Johnson, Raritan, NJ, United States; ^5^Department of Medicine, McMaster Immunology Research Centre, McMaster University, Hamilton, ON, Canada

**Keywords:** macrophage, cigarette smoke (CS), immunopathology, tissue remodelling, fibrogenesis

## Abstract

**Rationale:**

The accumulation of macrophages in the airways and the pulmonary interstitium is a hallmark of cigarette smoke-associated inflammation. Notably, pulmonary macrophages are not a homogenous population but consist of several subpopulations. To date, the manner in which cigarette smoke exposure affects the relative composition and functional capacity of macrophage subpopulations has not been elucidated.

**Methods:**

Using a whole-body cigarette smoke exposure system, we investigated the impact of cigarette smoke on macrophage subpopulations in C57BL/6 mice using flow cytometry-based approaches. Moreover, we used bromodeoxyuridine labelling plus *Il1a^-/-^* and *Il1r1^-/-^* mice to assess the relative contribution of local proliferation and monocyte recruitment to macrophage accumulation. To assess the functional consequences of altered macrophage subpopulations, we used a model of concurrent bleomycin-induced lung injury and cigarette smoke exposure to examine tissue remodelling processes.

**Main Results:**

Cigarette smoke exposure altered the composition of pulmonary macrophages increasing CD11b^+^ subpopulations including monocyte-derived alveolar macrophages (Mo-AM) as well as interstitial macrophages (IM)1, -2 and -3. The increase in CD11b^+^ subpopulations was observed at multiple cigarette smoke exposure timepoints. Bromodeoxyuridine labelling and studies in *Il1a^-/-^* mice demonstrated that increased Mo-AM and IM3 turnover in the lungs of cigarette smoke-exposed mice was IL-1α dependent. Compositional changes in macrophage subpopulations were associated with impaired induction of fibrogenesis including decreased α-smooth muscle actin positive cells following intratracheal bleomycin treatment. Mechanistically, *in vivo* and *ex vivo* assays demonstrated predominant macrophage M1 polarisation and reduced matrix metallopeptidase 9 activity in cigarette smoke-exposed mice.

**Conclusion:**

Cigarette smoke exposure modified the composition of pulmonary macrophage by expanding CD11b^+^ subpopulations. These compositional changes were associated with attenuated fibrogenesis, as well as predominant M1 polarisation and decreased fibrotic activity. Overall, these data suggest that cigarette smoke exposure altered the composition of pulmonary macrophage subpopulations contributing to impaired tissue remodelling.

## Introduction

A strong association has been shown between cigarette smoking and respiratory diseases such as, chronic obstructive pulmonary disease (COPD), lung cancer, and interstitial lung disease. Central to the pathogenesis of these cigarette smoke (CS)-associated respiratory diseases is the macrophage (1–3). Increased in the lungs following CS exposure (4, 5), macrophages perform a vital role in CS-induced inflammation (6). Of note, pulmonary macrophages consist of several subpopulations with independent and diverse functional roles (7–11). The composition and functional consequences of CS exposure on pulmonary macrophage subpopulations is yet to be elucidated.

Pulmonary macrophages can be stratified into two broad populations, alveolar macrophages (AM) and interstitial macrophages (IM). In mice, the first developmental wave occurs in the yolk sac on embryonic day E10-12 producing primitive AM (12, 13). The longevity of primitive AM is unclear; however, a second developmental wave arises from the foetal liver and enter the lungs by E12-16 (12, 13). These pre-AM enter the lumen postnatally (12, 13) and mature into long-lived tissue resident alveolar macrophages (Res-AM). These mature Res-AM are predominately self-maintained with limited contribution from circulating monocytes (14). A third AM subpopulation, monocyte-derived alveolar macrophages (Mo-AM), are recruited from the bone marrow after birth (14). Mo-AM share 99.9% of genes with Res-AM (15) but contribute to less than 5% of the alveolar population under homeostatic conditions (13). In addition to AM populations, IM are present and constitute approximately 20% of the pulmonary macrophage environment at steady state (13, 16). The majority of IM are produced postnatally in the bone marrow and populate the lung parenchyma throughout life (12, 17). Notably, a proportion of IM are derived from the yolk sac and are self-maintained similar to Res-AM populations (11). IM are further divided into three subpopulations based on CD11c and MHCII expression IM-1 - CD11c^Neg^MHCII^Neg^, IM–2 - CD11c^Neg^MHCII^Hi^, IM–3 - CD11c^Hi^MHCII^Hi^) (17). In total five pulmonary macrophage populations, Res-AM, Mo-AM plus IM1, -2 and -3 are detectable by flow cytometry-based techniques in mice.

The developmental origin of macrophage subpopulations is associated with differing function. At steady state, the phagocytic capacity is greatest in AM populations, followed by IM1, IM2 and poorest in IM3 (17). In contrast, IM populations are enriched for inflammatory mediators and monocyte-related genes distinct from AM (17). These observations have been replicated in COPD, wherein IM were found to be more proinflammatory, but less phagocytic, than AM counterparts (7). Macrophages are key in tissue remodelling processes and interstitial CX_3_CR1 mononuclear phagocytes, encompassing IM1, -2 and -3 populations (17), have been reported to promote CS-induced emphysema (18). Moreover, in pre-clinical pulmonary fibrosis models, aberrant tissue remodelling has been specifically associated with increased Mo-AM (9, 10, 19) and reduced IM1 (8). Given that macrophage subpopulations instigate inflammation that can lead to respiratory disease, further investigation of the impact of CS on individual macrophage subpopulations is warranted.

In this study, we investigated the impact of CS exposure on macrophage subpopulation composition and function. Using a whole-body CS exposure system, we assessed CS-induced alterations in macrophage populations, including CD11b^+^ subpopulations Mo-AM, IM1, -2 and -3, in the lungs. Moreover, we evaluated the impact of concurrent bleomycin treatment and CS exposure to understand macrophage subpopulation polarisation and ability to facilitate tissue remodelling. These data explored the impact of CS exposure on macrophage subpopulation composition, origin and function to offer insight into CS-associated respiratory disease.

## Materials and Methods

### Animals

6- to 8-week-old female C57BL/6 mice were purchased from Charles River Laboratories (PQ, Canada). Female IL-1α-deficient (*Il1a^-/-^*) mice were obtained from MiceCenter for Experimental Medicine and Systems Biology, University of Tokyo, Japan and bred in-house. Female IL-1R1-deficient (*Il1r1^-/-^*) mice (C57BL/6 background) with respective wild-type controls were obtained from The Jackson Laboratories (Maine, USA). Mice were subjected to a 12-hour light–dark cycle and had ad libitum access to food and water and housed under specific pathogen-free conditions. All experiments were approved by the Animal Research Ethics Board at McMaster University (#19-08-23).

### Experimental CS Exposure Model

Mice were exposed to twelve 3R4F reference cigarettes with filters removed (University of Kentucky, Lexington, USA) or room air (RA), twice daily for five days per week. Mice were exposed for up to 24-weeks using a whole-body CS exposure system SIU-48 (Promech Lab AB, Vintrie Sweden). Upon exposure completion, mice were euthanized by exsanguination and cardiac puncture.

### Tissue Processing for Flow Cytometric Analysis

Single cell suspensions were produced as follows. Mouse right middle, inferior, and post-caval lobes were enzymatically digested (150U/mL collagenase type I) for 1-hour shaking at 37°C. Bronchoalveolar lavage (BAL) was collected following 2x 500µL phosphate-buffered saline (PBS) washes of the single left lung lobe and BAL samples were then spun at 300 g for 5-minutes to pellet cells. To assess bone marrow, a single femur was removed and cells were flushed out using 5mL RPMI. Digested lung, flushed bone marrow, and harvested spleens were all crushed through 40µm mesh. Lung cells were treated with ACK lysis buffer (1mL, 1-minute) to lyse red blood cells. Approximately 80μL of blood was drawn using retro-orbital bleeding, lysed using 1x Red Blood Cell lysis buffer (#00-4333-57, eBioscience) and spun at 300 g for 5-minutes. All single cell suspensions were stained for flow cytometric analysis using antibodies shown in **Table S1**. Gating strategies are shown in **Figures S1A–D**. All samples were run on a BD LSRFortessa (BD Biosciences, ON, Canada).

### Bromodeoxyuridine Delivery

CS- or RA-exposed mice were intraperitoneally injected with bromodeoxyuridine (BrdU) for three consecutive days prior to sacrifice. 200μg on day -3 then subsequently 100μg on day -2 and -1 to assess macrophage turnover. BrdU incorporation into cells was assessed by flow cytometry.

### Experimental Pulmonary Fibrosis Model

Tissue remodelling was induced with a single intratracheal instillation of bleomycin (0.05U/mouse in a volume of 50µl sterile saline) by oropharyngeal administration. Control animals received 50µl vehicle alone. Weights of animals were monitored regularly, and tissue harvest was conducted 7 (early inflammatory phase) or 21 (fibrotic phase) days following bleomycin intubation. Lung function assessments were conducted using a flexiVent^®^ mechanical respirator according to manufacturer’s protocol at day 21 (flexiVent^®^, SCIREQ, Montreal, PQ, Canada) (20).

### Isolation and Stimulation of Lung CD45^+^ Adhered Cells

CD45^+^ cells from lung single-cell suspensions were positively selected using mouse CD45 microbeads and LS columns (#130-052-301 and #130-042-401, Miltenyi Biotech) and cultured on a flat 96-well plate for 90-minutes. Non-adherent cells were stringently washed with PBS before remaining adherent cells were treated for 24-hours with recombinant murine transforming growth factor beta 1 (TGF-β1) (30ng/mL; BioLegend, USA), IL-4 (20ng/mL; PeproTech, Canada) and IL-6 (5ng/mL; PeproTech, Canada). Macrophage “M2” polarisation was assessed by arginase activity in cell lysates, as described previously (21). Matrix metallopeptidase 9 (MMP9; #DY6718 R&D Systems, ON, Canada) ELISA, and CyQUANT™ lactose dehydrogenase (LDH; #C20301 ThermoFisher Scientific, ON, Canada) cytotoxicity assay, and soluble collagen assessed using sircol assay (Sircol™ Soluble Collagen Assay #CLRS1000, Biocolor, UK) were performed on cell culture supernatant.

### Tissue Collection for Protein and RNA Multiplex Analysis

Lung (right superior lobe) homogenate was processed using Bullet Blender 24 Gold (Next Advance, Troy NY USA) either in PBS or RLT lysis buffer (Qiagen, Valencia, CA, USA) for protein and RNA analysis respectively. Protein from lung homogenate supernatant was assessed using the Discovery Assay^®^ Mouse Cytokine Array/Chemokine Array 31-Plex (MD31; Eve Technologies Corp, Calgary, AB, Canada). Total RNA from RLT lysed lung homogenate was extracted using the RNeasy mini kit (Qiagen, Valencia, CA, USA, #74104). Following RNA integrity and quantity quality control the nCounter Elements system (NanoString Technologies, Seattle, WA, USA) was employed to quantify the expression levels of 25 mouse genes (**Table S2**).

### Lung Processing for Histopathology

Mouse single left lungs were inflated and fixed in 10% formalin for 24-hours prior to embedment in paraffin wax. A tissue microarray (**TMA**) was generated containing 5µm lung sections cut and stained on a Bond RX fully automated research Stainer (Leica Biosystems) (21). Immunohistochemistry (IHC) staining on lung serial sections was performed for hematoxylin & eosin (H&E), α-smooth muscle actin (α-SMA), and Masson’s trichrome blue (MTri). IHC stained-microscope slides were digitalised using an Olympus VS120-L100-W slide scanner at a 20× magnification. HALO™ Image Analysis Software (Halo Plus 3.2, Indica Labs) was used to quantify α-SMA and MTri stain in the lung parenchyma following the exclusion of airways and blood vessels using the Multiclass IHC (v3.0) and Classifier modules as previously described (22).

### Statistical Analysis

Results expressed as mean ± standard error of the mean. Graphs and statistical tests were performed using GraphPad Prism 9.1 (GraphPad Software, Inc) and R (www.r-project.org). Two-way ANOVA followed by Tukey’s multiple comparisons test was used to determine significance when two concurrent variables were compared. Unpaired t test with Welch’s correction was used to assess significance between only two groups. A p < 0.05 was considered statistically significant. Data obtained form the NanoString profiling were preprocessed and normalised using nSolver 2.5 software (www.nanostring.com) using three housekeeping genes *Actb, Pgk1, and Ywhaz* plus negative and positive controls. 25 mouse genes (**Table S2**) were used to perform principal component analysis (PCA); using prcomp function from rgl package (23), hierarchical clustering (Euclidean distance, complete agglomeration method, using heatmap.2 function from gplots package (24) in R) and differential expression analysis using limma package (25) in R. P-values were adjusted using BH correction for multiple testing (26) and adjusted p < 0.05 was considered statistically significant.

## Results

### CD11b^+^ Macrophage Subpopulations Increase in the Lungs of CS-Exposed Mice

**Figure 1:**
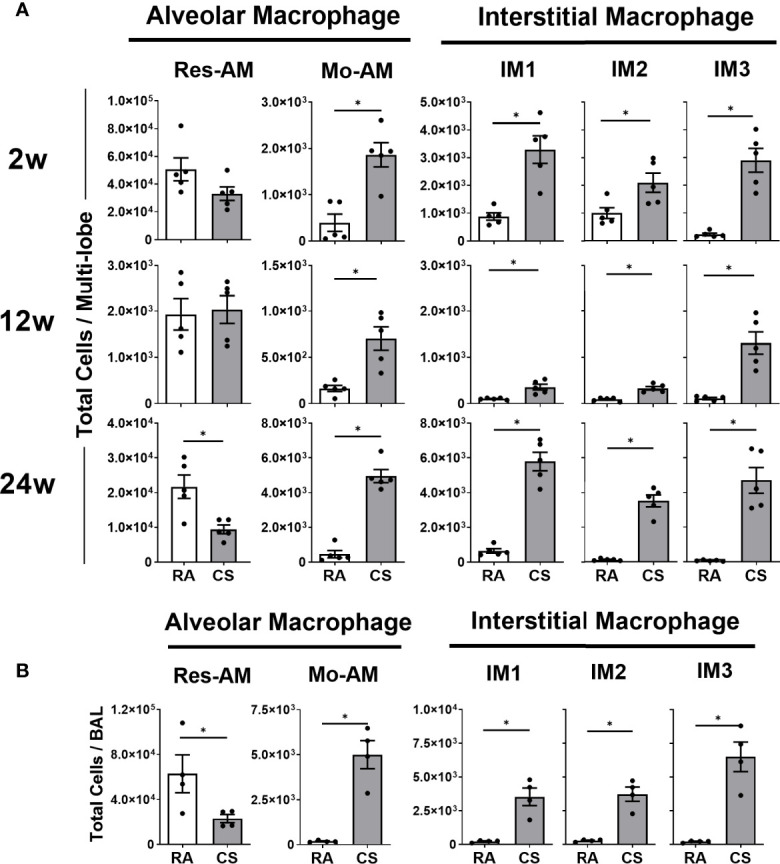
Cigarette smoke exposure alters pulmonary macrophage subpopulation composition expanding CD11b^+^ populations. Female C57BL/6 mice were RA or CS-exposed for 2- to 24-weeks. Data show total numbers of Res-AM, Mo-AM, IM1, IM2, and IM3 populations in **(A)** lung tissue and **(B)** bronchoalveolar lavage (BAL). Data show mean ± SEM, n = 5. Unpaired t test with Welch’s correction. RA, room air; CS, cigarette smoke. *p < 0.05.

CS is known to cause the expansion of the total lung macrophages (4, 5) but the impact on macrophage subpopulation diversity is not known. Using the nomenclature proposed by Gibbings et al. (17) (**Table 1**), we demonstrated an expansion of all CD11b^+^ populations including Mo-AM and IM1, -2 and – 3 at all CS timepoints (**Figure 1A**). Res-AM were unchanged at 2 and 12 weeks of CS but decreased at 24-weeks. SiglecF cell surface expression was highest in Res-AM with minimal expression in IM populations. To note, CS exposure decreased siglecF expression in Res-AM suggesting a downregulation of cell-cell interactions (**Figure S2A**). The alterations in macrophage subpopulation numbers were not driven by changes in total lung cellularity but represent changes to each subpopulation individually (**Figure S2B**). Independent of CS exposure length, each CD11b^+^ macrophage subpopulation was increased in CS-exposed mice.

**Table 1:** Cell surface expression of pulmonary macrophage subpopulations.

	Res-AM	Mo-AM	IM1	IM2	IM3
**CD64/MertK**	+	+	+	+	+
**CD11c**	+	+	–	–	+
**CD11b**	–	+	+	+	+
**SiglecF**	+	+/-	–	–	–
**MHCII**	+/-	–	–	+	+

Expression profile for macrophage subpopulations, based on Gibbings et al., used for flow cytometry analysis. Cells express marker (+), cells do not express (-), and cells have a spectrum of expression (-/+).

The size and location of macrophages has been associated with phagocytic and inflammatory differences (7). In this study, CD11b^+^ populations, Mo-AM (16.0%), IM1 (8.2%), and IM3 (7.1%), decreased in size at 12 weeks of CS (**Figure S2C**), corresponding to clinical observations of expanded small macrophages in COPD lung sections (7). We further assessed the presence of macrophage subpopulations in the BAL as a measure of macrophage location within the airways. While Res-AM decreased, each CD11b^+^ subpopulation, including all IM, were increased in the BAL of CS-exposed mice at 8 weeks of CS (**Figure 1B**). Clinically, airway macrophages are reported to be lipid-laden, and thus more granular (27). In our model, Res-AM became 197.9% and CD11b^+^ subpopulations 121.5-150.1% more granular than respective RA populations in the BAL (**Figure S2D**). Overall, we observed increased number of small, more granular, CD11b^+^ macrophages subpopulations following CS exposure.

### Increased CD11b^+^ Macrophage Turnover in CS-Exposed Mice

**Figure 2:**
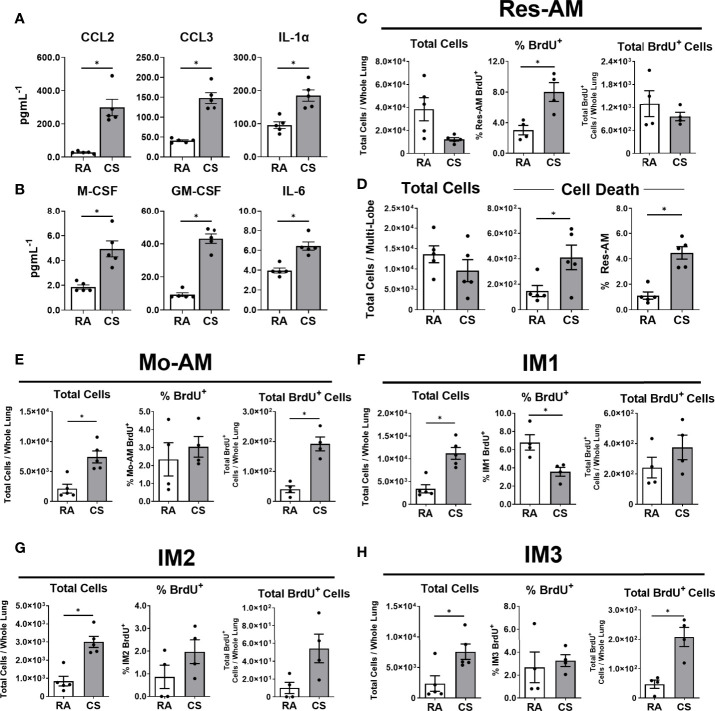
CD11b^+^ macrophages are recruited to the lung during cigarette smoke exposure. Female C57BL/6 mice were RA or CS-exposed for 12-weeks. Lung homogenate **(A)** immune mediators (CCL2, CCL3, and IL-1α) and **(B)** monocyte differentiation and macrophage survival factors (M-CSF, GM-CSF, and IL-6). BrdU was used to assess macrophage subpopulation turnover in the lung at 6-weeks CS. Data show **(C)** total cells, % BrdU^+^ and total BrdU^+^ Res-AM and **(D)** total cells, total dead and % dead Res-AM. Shown in **(E)** Mo-AM, **(F)** IM1, **(G)** IM2, and **(H)** IM3 are total cells, % positive and total BrdU^+^ cells. Data show mean ± SEM, n = 4 - 5. Unpaired t test with Welch’s correction. RA, room air; CS, cigarette smoke. *p < 0.05

To elucidate mechanisms contributing to increased CD11b^+^ macrophage populations, we assessed immune mediators associated with macrophage recruitment and survival in the lung homogenate. At 12 weeks of CS exposure myeloid chemoattractants CCL2 and CCL3 increased, as well as IL-1α (**Figure 2A**). IL-1α has previously been associated with CS-mediated myeloid cell recruitment (6, 28). Next, we measured immune mediators associated with monocyte differentiation and macrophage survival, M-CSF, GM-CSF and IL-6, all of which increased in the lung tissue of CS-exposed mice (**Figure 2B**). Immune mediators associated with macrophage recruitment and survival were increased in CS-exposed mice.

To understand pulmonary lung dynamics, we assessed macrophage subpopulation turnover using thymidine analogue BrdU. BrdU uptake can be used as a surrogate measure of cell turnover and the balance between local proliferation and cell recruitment. While the total Res-AM trended to decrease following CS exposure the percentage of Res-AM positive for BrdU increased (**Figure 2C**). Of note, the decreasing trend in total Res-AM was associated with increased cell death in CS-exposed mice as measured by flow cytometry Live/Dead positive staining (**Figure 2D**). Taken together, this suggests Res-AM proliferated in response to CS exposure to refill the environmental niche opened by increased Res-AM cell death. In contrast, CD11b^+^ populations had proportionally equal or fewer percentage BrdU^+^ cells, but increased total BrdU^+^ cells, following CS exposure (**Figures 2E–H**). The fixed percentage of BrdU positive cells indicated a stable rate of BrdU incorporation. Consequently, the increase in total BrdU^+^ Mo-AM and IM3 likely reflected a predominant recruitment of cells to the lungs. Total lung cell numbers were equivalent in RA and CS mice suggesting that changes were reflective of compositional shifts within the lung. Overall, CS was associated with Res-AM proliferation and a recruitment of Mo-AM and IM3 cells to the lung.

### Monocytes and Macrophage-Lineage Bone Marrow Progenitor Cells Are Transiently Decreased at 12 Weeks of CS Exposure

**Figure 3:**
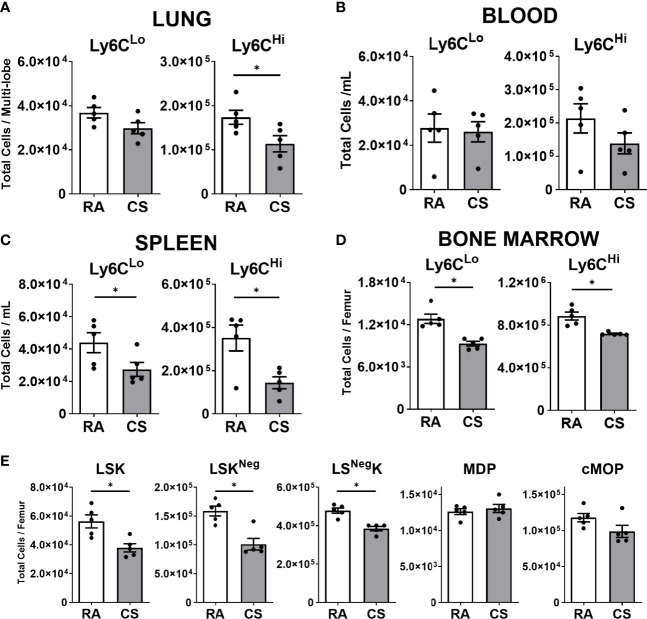
Macrophage progenitor cells are transiently decreased at 12-weeks of cigarette smoke exposure. Female C57BL/6 mice were RA or CS-exposed for 12-weeks. Data show total numbers of Ly6C^Lo^ and Ly6C^Hi^ monocyte populations in **(A)** lungs, **(B)** blood, **(C)** spleen, and **(D)** bone marrow. Shown in **(E)** total numbers of macrophage progenitor cells in the bone marrow (lineage-negative Sca1 c-Kit (LSK), monocyte-macrophage dendritic cell progenitor (MDP), common monocyte progenitor (cMoP)). Data show mean ± SEM, n = 5. Unpaired t test with Welch’s correction. RA, room air; CS, cigarette smoke. *p < 0.05

Given the expansion of CD11b^+^ populations including Mo-AM and IM3 in the lung, we next assessed the impact of CS on lung, blood, spleen, and bone marrow monocytes. In the lung, we observed that Ly6C^Lo^ monocyte populations, primed for macrophage differentiation, were not altered by CS exposure at 12-weeks. In contrast, classical inflammatory Ly6C^Hi^ monocytes were reduced by CS (**Figure 3A**). There was no change in either monocyte population in the circulation (**Figure 3B**). In the spleen, a known monocyte reservoir (29), CS resulted in decreased total spleen cells (**Figure S3A**) and consequently both monocyte populations (**Figure 3C**). CS exposure decreased both monocyte populations in the bone marrow at 12-weeks (**Figure 3D**). No changes in either monocyte population were observed in any tissue at 2- and 24-weeks CS exposure, with the exception of Ly6C^Lo^ monocytes, which were decreased following 2 weeks of CS exposure in the bone marrow (**Figures S3B–E**).

Next, we assessed macrophage progenitor cells in the bone marrow. The linearity of commitment toward cell terminal differentiation in order is Lineage^Neg^Sca1^+^c-Kit^+^(LSK), monocyte-macrophage dendritic cell progenitor (MDP), common monocyte progenitor (cMoP) and finally monocyte populations (30–32). We observed all LSK progenitor cell populations were decreased in CS-exposed mice at 12 weeks (**Figures 3E**, **S3F**). To note, more committed progenitor MDP and cMoP populations were unchanged by CS exposure (**Figure 3E**). CS exposure was associated with a transient reduction in lung, spleen and bone marrow monocyte and macrophage progenitor populations at 12 weeks of CS exposure.

### CS-Exposed Pulmonary Macrophage Expansion Is IL-1α Dependent

**Figure 4:**
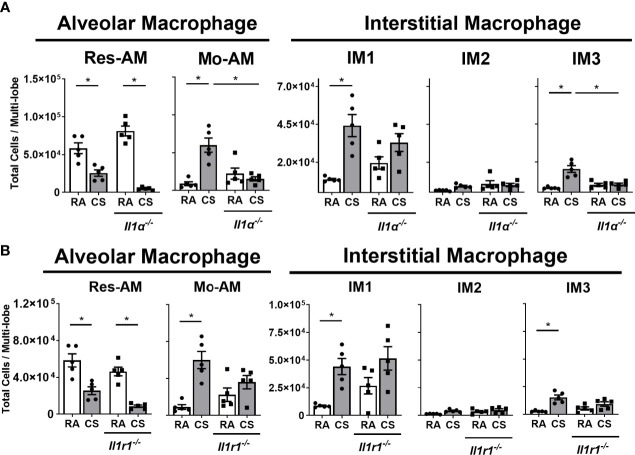
Macrophage expansion in cigarette smoke-exposed lung is IL-1α dependent. Data show total lung Res-AM, Mo-AM, IM1, -2 and -3 populations in **(A)** *Il1a^-/-^* and **(B)** *Il1r1^-/-^* mice compared to C57BL6 wild type control mice at 8-weeks CS. Data show mean ± SEM, n = 5. Two-way ANOVA with Tukey’s multi-comparison test. RA, room air; CS, cigarette smoke. *p < 0.05

The IL-1α axis is critical in the recruitment of myeloid cells to the lung following CS exposure (6, 33, 34). Consequently, we assessed the impact of IL-1α and IL1-R1 deficiency on macrophage subpopulations composition in CS-exposed mice. Res-AM decreased in both *Il1a^-/-^* and *Il1r1^-/-^* mice following CS exposure (**Figures 4A, B**). Mo-AM and IM3, which were the predominant subpopulations increased in total BrdU^+^ cells, had attenuated expansion in *Il1a^-/-^* mice following CS exposure (**Figure 4A**). There was no change in Mo-AM or IM3 populations in *Il1r1^-/-^* mice following CS exposure (**Figure 4B**). Moreover, IL-1α- and IL1-R1-deficiency was not associated with changes in IM1 or IM2 populations (**Figures 4A, B**). Cell number changes were not driven by differences in total cell number (**Figures S4A, B**). Thus, CS was associated with IL-1α dependent expansion of Mo-AM, IM3, and Res-AMs.

### CS Exposure Skews Macrophage Subpopulation Composition During Impaired Bleomycin-Induced Tissue Remodelling

**Figure 5:**
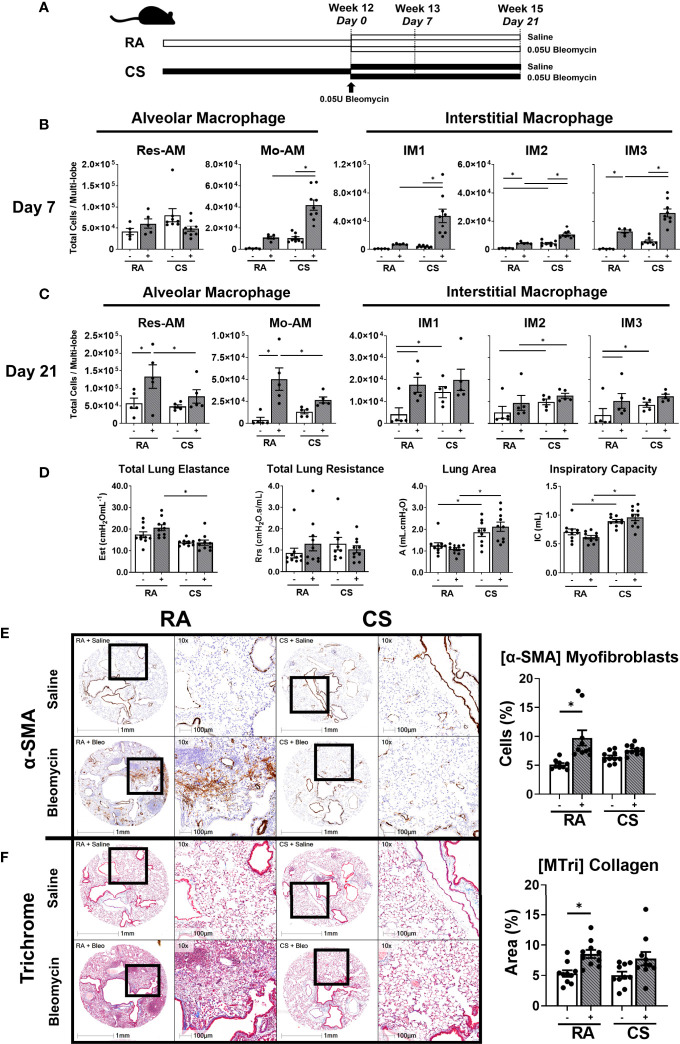
Skewed macrophage subpopulation composition is associated with decreased fibrogenesis. **(A)** Schematic of CS exposure with bleomycin instillation. C57BL/6 female mice were administered bleomycin (0.05U/mouse) (grey bars) or saline control (open bars) following 12-weeks of RA or CS exposure. Res-AM, Mo-AM, IM1, IM2, and IM3 populations in lung tissue following **(B)** 7 days or **(C)** 21 days of bleomycin administration. **(D)** Functional parameters derived from the pressure-driven pressure–volume loops included: total lung elastance, total lung resistance, lung area (atelectasis) and inspiration capacity measured at day 21. Data show representative image and HALO quantification for **(E)** α-SMA and **(F)** Masson’s trichrome. Data show mean ± SEM, n = 5 - 10. Two-way ANOVA with Tukey’s multi-comparison test. Est, elastance; Rrs, resistance; A, area; IC, inspiration capacity; RA, room air; CS, cigarette smoke. *p < 0.05. “-” represents saline administration. "+" represents 0.05U bleomycin administration.

Monocyte-derived macrophages, which contribute to the expanded CD11b^+^ macrophages in our model, have been shown to be necessary for fibrogenesis (9, 10, 19). Using a model of concurrent mild bleomycin-induced lung injury, we assessed the impact of CS on macrophage function at two timepoints, day 7 (pre-tissue remodelling and early inflammation) and day 21 (peak fibrogenesis and late inflammation) (**Figure 5A**) (35). While bleomycin-treated mice had equivalent weight loss regardless of exposure (**Figure S5A**), 3/5 (60%) and 4/5 (80%) myeloid lineage-related genes were enriched in CS-exposed compared to RA-exposed bleomycin-treated mice at day 7 and 21 respectively (**Tables S3** and **S4**). These transcriptional changes, including the upregulation of the CD11b-encoding gene *Itgam*, were likely related to the observed expansion of all CD11b^+^ subpopulations at day 7 in CS-exposed bleomycin-treated mice (**Figure 5B**). This compositional phenotype was not maintained in CS-exposed bleomycin-treated mice at day 21, with only IM2 remaining expanded (**Figure 5C**). In contrast, at day 21, Res-AM and Mo-AM populations were decreased and IM1/IM3 were equivalent in CS-exposed compared to RA-exposed bleomycin-treated mice (**Figure 5C**).

These alterations in macrophage composition at day 7 and 21 were associated impaired fibrogenesis by day 21 as determined by biomechanical, transcriptional and histological measurements. CS-exposed compared to RA-exposed bleomycin-treated mice had reduced total lung elastance (Est) but increased inspiratory capacity (IC) and lung area (A) (**Figure 5D**) at day 21. These physiological changes were associated with the downregulation of 5/9 (55%) fibrotic/wound healing genes at day 7 and 3/9 (33%) at day 21 in CS-exposed compared to RA-exposed bleomycin-treated mice (**Tables S3**, **S4**). Notably, overall transcriptional changes were sufficient to define experimental groups by unsupervised hierarchical clustering analysis at day 7 but not at day 21, where clustering was split based on CS status (**Figures S5B, C**). These altered transcriptional and lung biomechanics were associated with attenuated expansion of α-SMA^+^ cells in CS-exposed compared to RA-exposed mice (**Figure 5E**). Moreover, while the percent of collagenous area increased in RA-exposed bleomycin-treated mice compared to non-treated RA controls, collagen deposition was variable in CS-exposed bleomycin-treated mice and did not increase compared to controls (**Figure 5F**). Thus, trichrome staining suggests impaired collagen production in CS-exposed mice. Representative H&E stains are shown in (**Figure S5D**). Overall, these data suggest CS alters the transcriptional environment and macrophage composition at day 7 post bleomycin in a manner conductive to impaired fibrogenesis, as evidenced by decreased α-SMA^+^ myofibroblasts, elastance and collagen deposition at day 21.

### Macrophage Function and Polarisation Is Skewed by CS Exposure

**Figure 6:**
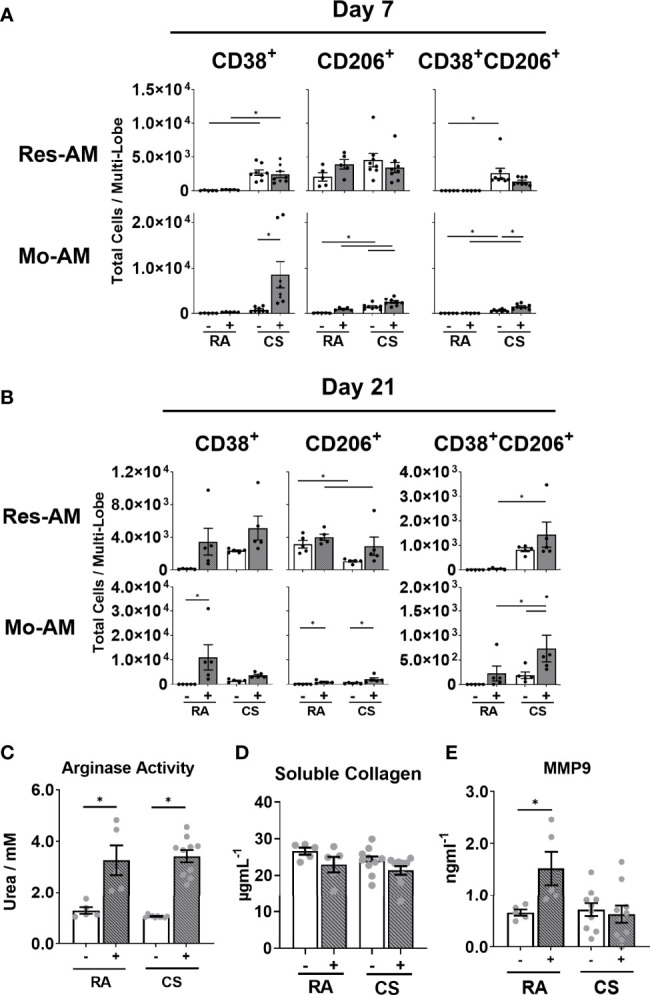
Macrophage subpopulation function and polarisation is altered by cigarette smoke exposure. C57BL/6 female mice were administered bleomycin (0.05U/mouse) (grey bars) or saline control (open bars) following 12-weeks of RA or CS exposure. Total number of Res-AM and Mo-AM expressing CD38, CD206 and CD38/CD206 in lung tissue following **(A)** 7 days or **(B)** 21 days post bleomycin administration. Adherent lung CD45^+^ cells isolated from 12-week RA- or CS-exposed C57BL/6 cell supernatant **(C)** urea production, **(D)** soluble collagen and **(E) **MMP9 release following TGF-β1, IL-4, IL-6 stimulation. Data show mean ± SEM, n= 5-10. Two-way ANOVA with Tukey’s multi-comparison test. RA, room air; CS, cigarette smoke. *p < 0.05. “-” represents saline administration **(A–E)**. "+" represents 0.05U bleomycin administration **(A, B)** or TGF-b1, IL-4, IL-6 administration **(C–E)**.

Given that we observed concurrent changes in macrophage composition and fibrotic outcome, we next sought to address whether CS specifically alters macrophage fibrogenic phenotype and function. M2-polarised, alternatively activated macrophages are proposed to contribute to tissue remodelling and fibrogenesis (21, 36). Consequently, using CD38 as a marker for M1-like and CD206 for M2-like macrophages, we assessed the polarisation state of each macrophage subpopulation at day 7 and 21 post bleomycin. While polarised macrophages comprised less than 30% of each subpopulation, of those cells polarised, CD38^+^ macrophages formed the greatest proportion at both day 7 and 21. CD38 expression was increased in all macrophage subpopulations of CS-exposed bleomycin-treated mice at day 7 (**Figures 6A**, **S6A**). By day 21, polarised macrophages remained predominately CD38^+^ but strikingly CD206^+^ Res-AM decreased in CS-exposed compared to RA-exposed bleomycin treated mice (**Figures 6B**, **S6B**). At both day 7 and day 21 CS-induced dual CD38^+^CD206^+^ expression, reflecting previous dual polarisation observations in CS only exposure models (37) (**Figures 6A, B**, **S6A, B**). Of note, no difference in total cell number was observed between bleomycin-treated groups at either day 7 or 21 (**Figures S6C, D**). To explore the impact of CS on macrophage function further, adherent lung CD45^+^ cells isolated following 12-weeks of CS or RA exposure were stimulated for 24-hours with a profibrotic cytokine mix (TGF-β1, IL-4, and IL-6). Comprising predominantly pulmonary macrophages, but not excluding monocytes or dendritic cells, we observed no difference in cell viability between experimental groups (**Figure S6E**). Arginase activity (as measured by urea production), a surrogate measure of alternatively-activated macrophage function, was equivalent in RA or CS-exposed cells following stimulation and demonstrating no loss in M2-like functionality (**Figure 6C**). In addition, while adherent CD45^+^ cells produced soluble collagen, CS exposure did not alter production and therefore contribute to changes in collagen deposition (**Figure 6D**). To note, the profibrotic cytokine mix induced MMP9, a peptidase implicated in CS-mediated epithelial-mesenchymal transition and myofibroblast development in fibrogenesis, in RA-derived cells. (**Figure 6E**) (38). This induction was not observed in CS-exposed cells treated with, or without, cytokine mix suggesting CS was sufficient to attenuate MMP9 release. In summary, CS exposure elicited a shift toward a M1-dominant phenotype and decreased MMP9 release.

## Discussion

Macrophages perform a central role in the pathogenesis of several CS-associated respiratory diseases including COPD and interstitial lung disease (1–3). However, the composition of pulmonary macrophage subpopulations following CS exposure is poorly understood. Using a mouse model of CS exposure, we showed increased CD11b^+^ macrophage populations, including Mo-AM and IM1, -2 and -3, in CS-exposed mice. The expansion of Mo-AM and IM3 populations was IL-1α dependent and was associated with a transient decrease in monocyte and progenitor populations in the bone marrow, spleen, and lungs, at 12 weeks of CS. These compositional changes were exacerbated in a model of bleomycin-induced lung injury. Moreover, CS exposure increased M1-polarised macrophages and was associated with impaired MMP9 release. Ultimately, these macrophage compositional and functional changes were associated with decreased fibrogenesis and impaired tissue remodelling in CS-exposed mice.

IM populations are reported to be located in the lung parenchyma and associated with vascular integrity and antigen presentation (8, 11). In CS-exposed mice, we observed all IM populations in the BAL. It is possible that the greater epithelial permeability in cigarette smokers (39, 40) enables greater recovery of IM from CS-exposed mice. To note, these cells likely do not reflect a transitioning IM cell into a Mo-AM as these populations are reported to have independent ontologies (10). Distinguishing IM and AM populations by histology is challenging due to the spectrum of cellular markers expressed which are shared between macrophage subpopulations. Further analysis is therefore warranted to determine whether IM1, -2 and -3 populations continue to reside in interstitial spaces upon CS exposure. The presence and the functional consequence of IM in the alveolar space warrants further investigation.

CS exposure caused a robust expansion of CD11b^+^ (Mo-AM, IM1, -2, -3) populations in the lung. These expanded CD11b^+^ populations have been previously reported to be derived from monocytes (10, 17). We observed transient decreases in macrophage/monocyte progenitor populations in the bone marrow and decreased monocytes in the lung and spleen following CS exposure. Moreover, we observed increased total BrdU^+^ Mo-AM and IM3 cell turnover in CS-exposed mice. It is therefore plausible these observations represent a CS-induced recruitment of monocytes which differentiate into Mo-AM and IM3 populations in the lung. However, targeted lineage tracing experiments are needed to confirm the ontogeny of these macrophage subpopulations in CS-exposed lungs. Res-AM numbers were unchanged by CS exposure with an equal balance between increased cell death and cell proliferation. This paradigm is supported by previous data that showed Res-AM have minimal postnatal recruitment and are self-maintained at steady state (12). Taken together, these data suggest that CS exposure promotes the expansion of CD11b^+^ populations, and specifically Mo-AM and IM3, altering pulmonary macrophage composition.

The IL-1α axis has been shown to be vital in CS-induced inflammation (6, 33, 34). In *Il1a^-/-^* mice, we observed reduced Mo-AM and IM3. Notably, these populations had increased numbers positive for BrdU which suggested increased cell turnover. Overall, these data propose an IL-1α dependent recruitment of Mo-AM and IM3 populations to CS-exposed lungs. Furthermore, Res-AM expansion was also IL-1α dependent whereas IM1 and IM2 expansion was not. We speculate the smaller contribution of IL-1α to IM1 and IM2 populations is a consequence a proportion of yolk sac-derived cells to IM1 and IM2 populations. IM1 and IM2 populations share a cellular phenotype with self-maintained and long-lived yolk sac-derived IM cells (11). Thus, we speculate long-lived yolk sac-derived IM cells are less dependent on IL-1α than foetal liver- or bone marrow-derived macrophage subpopulations.

Macrophages perform a vital role in the repair and regeneration of the tissue following damage (41). Using a model of bleomycin-induced lung injury, we observed attenuated tissue remodelling following CS exposure which was associated with decreased Res-AM and Mo-AM populations at peak fibrogenesis. Mo-AMs are necessary for fibrogenesis in mouse models of fibrosis (9, 10); thus, a reduction in Mo-AM in CS-exposed compared to RA-exposed bleomycin-treated mice may represent an impairment in fibrogenesis in our model. In addition, IM1 which have been reported to protect against fibrosis (8), were expanded in CS-exposed mice and therefore may also contribute to an anti-fibrotic environment. Notably, CS exposure induced phenotypically a greater number of M1-like compared to M2-like-polarised macrophages at both day 7 and 21 regardless of subpopulation. Lung transcriptional changes in fibrotic/wound healing and M2-realted genes were most discordant at the day 7 timepoint between bleomycin-treated groups. This phenotypic shift was not associated with any difference in arginase activity suggesting macrophages remain capable of M2-like functional activity despite the increase in M1-like cells. Detailed investigation of the impact of CS on each macrophage subpopulation’s function is needed to elucidate the precise mechanisms altered and that contribute to impaired tissue remodelling. Taken together, these data suggest that CS exposure alters pulmonary macrophage composition and polarisation, decreasing the accumulation of profibrotic macrophages early in fibrogenesis progression leading to an impaired tissue remodelling phenotype by day 21.

Pressure–volume loops are used routinely to assess elastance as a measure of elastic stiffness in mouse models of bleomycin. At day 21, we observed no difference in elastance between RA-exposed saline-treated and RA-exposed mice treated with 0.05U/mouse bleomycin, despite increased α-SMA^+^ cells and collagen deposition. This phenomenon has previously been observed in mouse models of low bleomycin (0.04U/mouse) administration with higher bleomycin (0.06U/mouse) doses required to induce significant statistical differences in elastance (42). These data suggest that histological measurements are more sensitive to bleomycin insult and that higher bleomycin doses are required to observe physiological differences in RA-exposed mice. Notably, combined CS and bleomycin exposure was sufficient to decrease the lung elastic stiffness compared to RA-exposed bleomycin-treated mice at day 21. CS has previously shown to be associated with increased lung compliance (decreased elastance) (43). However, α-SMA^+^ cells and collagen deposition were not significantly altered between RA- and CS-exposed bleomycin-treated animals despite changes in elastance. These data suggest that CS has a multifactorial impact on lung elastance impacting both extracellular matrix components as well as surface tension *via* decreases in lung surfactant (44) which together result in CS-related decreased elastic stiffness. Notably, inspiration capacity and lung area (atelectasis) were also increased in CS-exposed mice demonstrating CS aberrant impact on lung physiology. Given CS decreased lung elastance as well as impairing α-SMA^+^ cell induction, studies using escalating bleomycin doses are needed to assess the impact of CS on histological and biomechanical parameters leading to tissue remodelling.

CS is well-known to compromise tissue repair through such processes as impaired myofibroblast differentiation (45). Specifically, MMP9 has been shown to contribute to TGF-β production (46) and epithelial-mesenchymal transition (38, 47), processes central to myofibroblast differentiation (48). We observed impaired MMP9 expression in isolated adherent lung CD45^+^ cells from 12-week CS-exposed mice. Notably, the impact of CS on MMP9 regulatory molecules in this two-hit model are not known. Given the autoregulatory role of transcriptional regulators such as NFκB following chronic activation (49), CS exposure may lead to a self-limiting inflammatory response which downregulates MMP9 release. Decreased MMP9 release in CS-exposed adherent CD45^+^ cells may contribute to impaired, or delayed, α-SMA^+^ myofibroblast expansion. It is plausible the CS-mediated reduction in α-SMA^+^ myofibroblasts, a major collagen producing cell (50), is critical in the impaired tissue remodelling and repair response observed in this bleomycin-induced lung injury model. However, these data do not show a casual link between MMP9 release and myofibroblast expansion. Further investigation of macrophage function is needed to elucidate mechanisms that contribute to CS-induced impaired myofibroblast expansion and aberrant tissue remodelling.

In summary, we showed that CS altered pulmonary macrophage composition, increasing CD11b^+^ subpopulations, including Mo-AM and IM1-2 and -3, at multiple CS exposure timepoints. The expansion of Mo-AM and IM3 was dependent on IL-1α and likely reflective of increased cell recruitment. Compositional changes were associated with predominately M1-like macrophages, attenuated MMP9 release and decreased fibrogenesis in a model of bleomycin-induced lung injury. Taken together, these data propose that CS exposure skews pulmonary macrophage subpopulation composition and function predisposing the host to impaired tissue remodelling following lung injury.

## Data Availability Statement

The original contributions presented in the study are included in the article/**Supplementary Material**. Further inquiries can be directed to the corresponding author.

## Ethics Statement

The animal study was reviewed and approved by Animal Research Ethics Board at McMaster University.

## Author Contributions

Conception and design: SC, OM, MS, KA. Performed experiments: SC, OM, DT, JM, SR, MF, PW, AR, AH, FB. Analysis and interpretations: SC, OM, AD-G, MS, KA. Drafting the manuscript: SC, OM. Provided resources: CS, MS, KA. Edited and revised manuscript: SC, OM, DT, JM, SR, MF, FB, MS, KA. All authors contributed to the article and approved the submitted version.

## Funding

This work was funded by the Canadian Institutes of Health Research (PJT-159792), RespiVert Ltd. part of Janssen Pharmaceuticals, the Canadian Pulmonary Fibrosis Foundation, and the Lung Health Foundation. The funders were not involved in the study design, collection, analysis, interpretation of data, the writing of this article or the decision to submit it for publication.

## Conflict of Interest

As of January 2020, MS is an employee of CSL Behring AG. CS was employed by company Johnson and Johnson. KA reports grants from Alkermes, Prometic, GSK, Canadian Institute for Health Research, Pharmaxis, Indalo, Unity Biotechnology, Canadian Pulmonary Fibrosis Association, Collaborative Health Research Projects, Pieris Pharmaceuticals, Bold Therapeutics, Pliant, CSL Behring and grants and personal fees from Boehringer Ingelheim outside of the submitted work.

The remaining authors declare that the research was conducted in the absence of any commercial or financial relationships that could be construed as a potential conflict of interest.

## Publisher’s Note

All claims expressed in this article are solely those of the authors and do not necessarily represent those of their affiliated organizations, or those of the publisher, the editors and the reviewers. Any product that may be evaluated in this article, or claim that may be made by its manufacturer, is not guaranteed or endorsed by the publisher.

## Supplementary Material

Supplementary Table 1 Flow cytometry panel. A panel of surface and intracellular markers to examine the myeloid cells in mouse lung, blood, spleen and bone marrow.

Supplementary Table 2 NanoString custom designed fibrogenesis panel. A panel to assess the expression of genes related to wound healing/fibrogenesis, monocytes/macrophages, M1 and M2 macrophage polarisation.

Supplementary Table 3 mRNA differential expression of fibrogenesis-associated genes at day 7 post bleomycin. Fold change of genes related to wound healing/fibrogenesis, myeloid, M1 and M2 macrophage polarisation. Blank wells represent no significant differential expression between groups. Shown are genes significantly differentially expressed at day 7. Limma package, R. RA, room air; CS, cigarette smoke; FC, fold change.

Supplementary Table 4 mRNA differential expression of fibrogenesis-associated genes at day 21 post bleomycin. Fold change of genes related to wound healing/fibrogenesis, myeloid, M1 and M2 macrophage polarisation. Blank wells represent no significant differential expression between groups. Shown are genes significantly differentially expressed at day 21. Limma package, R. RA, room air; CS, cigarette smoke; FC, fold change.

Supplementary Figure 1 Monocyte and macrophage subpopulations gating strategies. **(A)** Lungs were enzymatically digested and stained for flow cytometry. Diagrams showing gating strategies used to isolate total macrophage populations identified as Live autofluorescent (FITC) +/-Lineage^Neg^CD45^+^MertK^+^CD64^+^. Macrophage lineage includes CD3, CD19, NK1.1, EpCAM and Ly6G. We further distinguished CD11c^+^CD11b^Neg^SiglecF^Hi^ resident alveolar macrophages (Res-AM), CD11c^+^CD11b^+^MHCII^Neg^ monocyte-derived alveolar macrophages (Mo-AM), CD11c^Neg^CD11b^+^MHCII^Neg^ interstitial macrophage 1 (IM1), CD11c^Neg^CD11b^+^MHCII^+^ (IM2), CD11c^+^CD11b^+^MHCII^+^ (IM3) plus Ly6C^Lo^, Ly6C^Hi^ monocyte subsets. Blood **(B)** and spleen **(C)** monocyte populations were determined as CD45^+^Ly6G^Neg^CD11b^+^CD115^+^ then Ly6C^Lo^ and Ly6C^Hi^. **(D)** Bone marrow monocytes and progenitors were defined as CD45^+^Lineage^Neg^. Subsequently monocytes defined as CD11b^+^CD115^+^CD117^Neg^CD135^Neg^ then Ly6C^Lo^ and Ly6C^Hi^. Bone marrow lineage includes CD3, CD19, NK1.1, CD90.2 and Ly6G. Myeloid progenitors were defined as CD115^+^CD117^+^CD135^Neg^CD11b^Neg^Ly6C^Hi^ common monocyte progenitor (cMoP), CD115^+^CD117^+^CD135^+^CD11b^Neg^Ly6C^Neg^ monocyte-macrophage dendritic cell progenitor (MDP) plus CD115^Neg^CD117^+^Sca1^Hi^ Sca1 c-Kit (LSK), CD115^Neg^CD117^+^Sca1^Neg^ LS^Neg^K and CD115^Neg^CD117^Neg^Sca1^+^ LSK^Neg^. Fluorescence minus one was used to gate each of the population of interest.

Supplementary Figure 2 Macrophage size and granularity following cigarette smoke exposure. **(A)** Median fluorescent intensity of siglecF expression for all macrophage subpopulations at 12 weeks CS exposure. Data show total numbers of **(B)** lung (2-, 12-, 24-weeks) and BAL (8-weeks) cells following CS exposure. **(C)** Res-AM, Mo-AM, IM1, IM2, and IM3 population size measured by forward scatter (FSC). **(D)** Res-AM, Mo-AM, IM1, IM2, and IM3 population granularity measured by side scatter (SSC). Data show mean ± SEM, n = 5. Unpaired t test with Welch’s correction. MFI, median fluorescent intensity; RA, room air; CS, cigarette smoke.

Supplementary Figure 3 Progenitor and monocyte populations at 2, 12 and 24-weeks following cigarette smoke exposure. Female C57BL/6 mice were RA or CS-exposed for 2, 12, or 24-weeks. Data show total cell numbers in **(A)** lung, blood, spleen, and bone marrow. Data also show total numbers of Ly6C^Lo^ and Ly6C^Hi^ monocyte populations in **(B)** lung, **(C)** blood, **(D)** spleen, and **(E)** bone marrow and **(F)** total numbers of macrophage progenitor cells in the bone marrow (lineage-negative Sca1 c-Kit (LSK), monocyte-macrophage dendritic cell progenitor (MDP), common monocyte progenitor (cMoP)). Data show mean ± SEM, n= 4 - 5. Unpaired t test with Welch’s correction. RA, room air; CS, cigarette smoke.

Supplementary Figure 4 Pulmonary macrophages are expanded *via* IL1α during cigarette smoke exposure. Female C57BL/6, *Il1a^-/-^* and *Il1r1^-/-^* mice were RA or CS-exposed for 8-weeks. **(A)** Data show total cell number in **(A)** *Il1a^-/-^* and **(B)** *Il1r1^-/-^* mice plus C57BL6 wildtype controls. Data show mean ± SEM, n = 5. Two-way ANOVA with Tukey’s multi-comparison test. RA, room air; CS, cigarette smoke.

Supplementary Figure 5 Fibrotic measurements at day 21 of bleomycin instillation. C57BL/6 female mice were administered bleomycin (0.05U/mouse) or control saline following 12-weeks of RA or CS exposure. Mice were monitored 21 days prior to flexiVent^®^ lung measurements and tissue harvest. **(A)** Body weight was measured daily and shown as percentage change in body weight. Principal component analysis and heatmaps defined by 25 mouse genes from lung homogenate assessed by NanoString at **(B)** day 7 and **(C)** day 21. **(D)** Representative images for H&E-stained sections. Data show mean ± SEM, n= 4-10. Two-way ANOVA with Tukey’s multi-comparison test. RA, room air; CS, cigarette smoke.

Supplementary Figure 6 CD38^+^ macrophages are increased in cigarette smoke-exposed bleomycin-treated mice. C57BL/6 female mice were administered bleomycin (0.05U/mouse) or control saline following 12-weeks of RA or CS exposure. Graphs demonstrate total numbers of IM1, IM2, and IM3 populations expressing CD38, CD206 and CD38/CD206 in lung tissue following **(A)** 7 days or **(B)** 21 days of bleomycin administration. Total lung cell counts **(C)** day 7 and **(D)** day 21. Adherent lung CD45^+^ lactose dehydrogenase release in *ex vivo* TGF-β1, IL-6 and IL-4 stimulated cell supernatant from **(E)** 12-week room air (RA)- or cigarette smoke (CS)-exposed mice. Shown mean ± SEM, n = 4 - 10. Two-way ANOVA with Tukey’s multi-comparison test. RA, room air; CS, cigarette smoke.

## Abbreviations

α-SMA, alpha-Smooth muscle actin; AM, Alveolar macrophage; BrdU, Bromodeoxyuridine; COPD, Chronic obstructive pulmonary disease; cMoP, Common monocyte progenitor; CS, Cigarette smoke; CSE, Cigarette smoke extract; E, Embryonic day; H&E, Hematoxylin & eosin; IHC, Immunohistochemistry; IM, Interstitial macrophage; LDH, Lactose dehydrogenase; LSK, Lineage negative Sca1^+^c-Kit^+^; MMP9, Matrix metallopeptidase 9; Mo-AM, Monocyte-derived alveolar macrophages; MDP, Monocyte-macrophage dendritic cell progenitor; PBS, Phosphate-buffered saline; PCA, Principal component analysis; Res-AM, Resident alveolar macrophage; RA, Room air; TMA, Tissue microarray; TGF-β, 1Transforming growth factor beta 1.
