# Supplementary material for: Increased Monocyte-Derived CD11b+ Macrophage Subpopulations Following Cigarette Smoke Exposure Are Associated With Impaired Bleomycin-Induced Tissue Remodelling
Source: Front Immunol. 2021 Sep 16;12:740330. doi: 10.3389/fimmu.2021.740330 (PMC8481926; doi:10.3389/fimmu.2021.740330)
Supplement: Supplementary Table 1 — Flow cytometry panel. A panel of surface and intracellular markers to examine the myeloid cells in mouse lung, blood, spleen and bone marrow. [file Table_1.docx]

| Antibody Specificity | Conjugate | | Clone | | Supplier | |  |
| --- | --- | --- | --- | --- | --- | --- | --- |
| CD3 | APCeFlour780 | | 17A2 | | eBioscience | |  |
| CD11b | Pe Dazzle 594 | | M1/70 | | Biolegend | |  |
| CD11c | BV650 | | N418 | | Biolegend | |  |
| CD19 | APCeFlour780 | | eBio1D3 | | eBioscience | |  |
| CD24 | BV421 | | M1/69 | | Biolegend | |  |
| CD45 | AlexaFluor700 | | 30-F11 | | Biolegend | |  |
| CD64 | PeCy7 | | X54-5/71 | | Biolegend | |  |
| EpCAM | APC Cy7 | | G8.8 | | Biolegend | |  |
| LIVE/DEAD^TM^ | Fixable Yellow Stain | |  | | Thermo Fisher Scientific | |  |
| Ly6C | BV711 | | HK1.4 | | Biolegend | |  |
| Ly6G | APC Cy7 | | 1A8 | | Biolegend | |  |
| MerTk | APC | | 2B10C42 | | Biolegend | |  |
| MHCII | | PerCp Cy5.5 | | M5/114.15.2 | | Biolegend | |
| NK1.1 | | APC Cy7 | | PK136 | | Biolegend | |
| SiglecF | | Pe | | E50-2440 | | BD Pharmingen | |
| SiglecF | | BV421 | | S17007L | | Biolegend | |
| CD38 | | Pe | | 90 | | Biolegend | |
| CD206 | | BV785 | | C068C2 | | Biolegend | |
| CD115 | | BV421 | | AF598 | | Biolegend | |
| CD115 | | PE/Cy7 | | AF598 | | Biolegend | |
| CD90.2 | | APC Cy7 | | 30-H12 | | Biolegend | |
| CD117 | | APC | | 2B8 | | Thermo Fisher Scientific | |
| CD135 | | BV421 | | A2F10 | | Biolegend | |
| Sca1 | | BV650 | | D7 | | Biolegend | |
| BrdU | | APC | |  | | BD Biosciences | |

*Table S1*
