# Supplementary material for: Increased Monocyte-Derived CD11b+ Macrophage Subpopulations Following Cigarette Smoke Exposure Are Associated With Impaired Bleomycin-Induced Tissue Remodelling
Source: Front Immunol. 2021 Sep 16;12:740330. doi: 10.3389/fimmu.2021.740330 (PMC8481926; doi:10.3389/fimmu.2021.740330)
Supplement: Supplementary Table 2 — NanoString custom designed fibrogenesis panel. A panel to assess the expression of genes related to wound healing/fibrogenesis, monocytes/macrophages, M1 and M2 macrophage polarisation. [file Table_2.docx]

| **Wound healing/Fibrogenesis** | **Myeloid** | **M1** | **M2** |
| --- | --- | --- | --- |
| *Fgf2* | *Itgam* | *Nos2* | *Arg1* |
| *Pdgfa* | *Itgax* | *Tnf* | *Mrc1* |
| *Tgfb1* | *Ccl2* | *Il1b* | *Il4ra* |
| *Lrrc32* | *Il-10* | *Il1a* | *Il6ra* |
| *Vegfa* | *Cxcl1* |  | *Osmr* |
| *Fn1* |  |  | *Il6* |
| *Col1a1* |  |  | *Osm* |
| *Col3a1* |  |  |  |
| *Timp1* |  |  |  |

*Table S2*
