# Supplementary material for: Increased Monocyte-Derived CD11b+ Macrophage Subpopulations Following Cigarette Smoke Exposure Are Associated With Impaired Bleomycin-Induced Tissue Remodelling
Source: Front Immunol. 2021 Sep 16;12:740330. doi: 10.3389/fimmu.2021.740330 (PMC8481926; doi:10.3389/fimmu.2021.740330)
Supplement: Supplementary Table 3 — mRNA differential expression of fibrogenesis-associated genes at day 7 post bleomycin. Fold change of genes related to wound healing/fibrogenesis, myeloid, M1 and M2 macrophage polarisation. Blank wells represent no significant differential expression between groups. Shown are genes significantly differentially expressed at day 7. Limma package, R. RA, room air; CS, cigarette smoke; FC, fold change. [file Table_3.docx]

|  |  | **RA Bleomycin**  **vs.**  **RA Saline** | | | | **CS Bleomycin**  **vs.**  **CS Saline** | | | | **CS Bleomycin**  **vs.**  **RA Bleomycin** | | | **CS Saline**  **vs.**  **RA Saline** | | | |
| --- | --- | --- | --- | --- | --- | --- | --- | --- | --- | --- | --- | --- | --- | --- | --- | --- |
|  |  | **FC** | **Adj. P value** | | **FC** | | **Adj. P value** | | **FC** | | | **Adj. P value** | | **FC** | | **Adj. P value** |
| **Fibrosis/wound healing** | *Fgf2* | 1.46 | | 8.9E-07 | | 1.18 | | 3.5E-03 | | -1.32 | 1.3E-05 | | - | | - | |
|  | *Pdgfa* | -1.33 | | 3.2E-04 | | - | | - | | 1.17 | 1.6E-02 | | - | | - | |
|  | *Tgfb1* | -1.11 | | 6.1E-03 | | -1.08 | | 3.3E-02 | | 1.11 | 3.0E-03 | | - | | - | |
|  | *Lrrc32* | - | | - | | -1.37 | | 2.9E-04 | | - | - | | - | | - | |
|  | *Vegfa* | -1.28 | | 1.8E-03 | | - | | - | | - | - | | -1.19 | | 2.3E-02 | |
|  | *Fn1* | 2.82 | | 1.1E-08 | | 1.84 | | 1.1E-05 | | -1.48 | 1.3E-03 | | - | | - | |
|  | *Col1a1* | 2.71 | | 1.8E-09 | | 2.13 | | 3.7E-08 | | -1.42 | 9.5E-04 | | - | | - | |
|  | *Col3a1* | 2.45 | | 2.5E-10 | | 2.16 | | 5.8E-10 | | -1.38 | 1.9E-04 | | -1.22 | | 2.8E-02 | |
|  | *Timp1* | 11.18 | | 9.1E-12 | | 3.73 | | 3.8E-08 | | -1.81 | 1.2E-03 | | 1.65 | | 1.4E-02 | |
| **Myeloid** | *Itgam* | - | | - | | 1.36 | | 6.3E-04 | | 1.83 | 2.7E-08 | | 1.44 | | 4.0E-04 | |
|  | *Itgax* | 1.34 | | 8.6E-03 | | - | | - | | 1.67 | 6.4E-06 | | 2.68 | | 1.8E-09 | |
|  | *Ccl2* | 11.55 | | 2.0E-10 | | 1.7 | | 1.4E-02 | | - | - | | 7.41 | | 5.6E-09 | |
|  | *Il10* | -3.67 | | 2.5E-03 | | -3.49 | | 1.6E-03 | | - | - | | - | | - | |
|  | *Cxcl1* | 2.36 | | 5.4E-03 | | - | | - | | 2.14 | 5.2E-03 | | 7.64 | | 2.9E-07 | |
| **M1** | *Nos2* | 1.81 | | 3.7E-06 | | 1.43 | | 6.4E-04 | | - |  | | - | |  | |
|  | *Tnf* | 1.72 | | 7.5E-04 | | - | | - | | 1.50 | 3.0E-03 | | 2.79 | | 2.3E-07 | |
|  | *Il1b* | -1.81 | | 5.4E-04 | | - | | - | | - |  | | -1.41 | | 3.3E-02 | |
|  | *Il1a* | - | | - | | - | | - | | 1.71 | 3.7E-06 | | 1.75 | | 1.8E-05 | |
| **M2** | *Arg1* | 4.53 | | 2.3E-07 | | 3.14 | | 4.1E-06 | | -1.76 | 5.4E-03 | | - | | - | |
|  | *Mrc1* | - | | - | | -1.35 | | 9.5E-04 | | - | - | | 1.55 | | 5.5E-05 | |
|  | *Il4ra* | 1.31 | | 5.7E-06 | | 1.21 | | 2.2E-04 | | -1.15 | 2.0E-03 | | - | | - | |
|  | *Il6ra* | -1.83 | | 1.3E-09 | | -1.58 | | 2.7E-08 | | - | - | | - | | - | |
|  | *Osmr* | 1.62 | | 1.8E-09 | | 1.12 | | 2.0E-02 | | - | - | | 1.34 | | 8.8E-06 | |
|  | *Il6* | 2.72 | | 3.7E-05 | | - | | - | | - | - | | 1.93 | | 3.5E-03 | |
|  | *Osm* | 1.39 | | 2.9E-03 | | - | | - | | - | - | | 1.40 | | 2.8E-03 | |

*Table S3*
