# Supplementary material for: Increased Monocyte-Derived CD11b+ Macrophage Subpopulations Following Cigarette Smoke Exposure Are Associated With Impaired Bleomycin-Induced Tissue Remodelling
Source: Front Immunol. 2021 Sep 16;12:740330. doi: 10.3389/fimmu.2021.740330 (PMC8481926; doi:10.3389/fimmu.2021.740330)
Supplement: Supplementary Table 4 — mRNA differential expression of fibrogenesis-associated genes at day 21 post bleomycin. Fold change of genes related to wound healing/fibrogenesis, myeloid, M1 and M2 macrophage polarisation. Blank wells represent no significant differential expression between groups. Shown are genes significantly differentially expressed at day 21. Limma package, R. RA, room air; CS, cigarette smoke; FC, fold change. [file Table_4.docx]

|  |  | **RA Bleomycin**  **vs.**  **RA Saline** | | | | **CS Bleomycin**  **vs.**  **CS Saline** | | | | **CS Bleomycin**  **vs.**  **RA Bleomycin** | | | **CS Saline**  **vs.**  **RA Saline** | | | |
| --- | --- | --- | --- | --- | --- | --- | --- | --- | --- | --- | --- | --- | --- | --- | --- | --- |
|  |  | **FC** | **Adj. P value** | | **FC** | | **Adj. P value** | | **FC** | | | **Adj. P value** | | **FC** | | **Adj. P value** |
| **Fibrosis/wound healing** | *Fgf2* | - | | - | | - | |  | | - | - | | - | | - | |
|  | *Pdgfa* | - | | - | | 1.17 | | 4.3E-02 | | - | - | | - | | - | |
|  | *Tgfb1* | - | | - | | - | | - | | - | - | | 1.17 | | 4.5E-03 | |
|  | *Lrrc32* | - | | - | | - | | - | | - | - | | - | | - | |
|  | *Vegfa* | - | | - | | - | | - | | - | - | | -1.45 | | 1.2E-03 | |
|  | *Fn1* | 1.29 | | 2.2E-02 | | - | | - | | -1.25 | 4.6E-02 | | - | | - | |
|  | *Col1a1* | 1.65 | | 9.3E-05 | | - | | - | | -1.44 | 2.1E-03 | | - | | - | |
|  | *Col3a1* | 2.15 | | 3.6E-06 | | - | | - | | -1.52 | 1.6E-03 | | - | | - | |
|  | *Timp1* | 1.69 | | 2.0E-02 | | - | | - | | - | - | | 2.14 | | 1.2E-03 | |
| **Myeloid** | *Itgam* | -1.27 | | 2.0E-02 | | - | | - | | 1.67 | 2.6E-05 | | 1.30 | | 1.1E-02 | |
|  | *Itgax* | 1.30 | | 4.0E-02 | | - | | - | | 1.91 | 2.5E-05 | | 2.52 | | 3.3E-07 | |
|  | *Ccl2* | - | | - | | - | | - | | 7.82 | 1.1E-08 | | 8.78 | | 8.5E-09 | |
|  | *Il10* | - | | - | | - | | - | | - |  | | 4.04 | | 3.7E-03 | |
|  | *Cxcl1* | - | | - | | - | | - | | 6.37 | 4.1E-08 | | 4.15 | | 1.6E-06 | |
| **M1** | *Nos2* | 2.06 | | 1.1E-03 | | - | | - | | - |  | | - | | - | |
|  | *Tnf* | - | | - | | - | | - | | 1.93 | 6.3E-05 | | 1.95 | | 5.5E-05 | |
|  | *Il1b* | -1.40 | | 2.0E-02 | | - | | - | | - |  | | -2.27 | | 6.4E-06 | |
|  | *Il1a* | - | | - | | - | | - | | 1.89 | 2.5E-05 | | 1.99 | | 9.9E-06 | |
| **M2** | *Arg1* | - | | - | | 1.59 | | 2.0E-02 | | - |  | | -2.09 | | 6.0E-04 | |
|  | *Mrc1* | - | | - | | - | | - | | 1.52 | 7.4E-04 | | 1.68 | | 6.3E-05 | |
|  | *Il4ra* | - | | - | | - | | - | | - | - | | - | | - | |
|  | *Il6ra* | -1.58 | | 1.2E-03 | | - | | - | | - | - | | -1.34 | | 3.0E-02 | |
|  | *Osmr* | - | | - | | - | | - | | - | - | | - | | - | |
|  | *Il6* | - | | - | | - | | - | | - | - | | - | | - | |
|  | *Osm* | - | | - | | - | | - | | - | - | | -1.44 | | 4.7E-03 | |

*Table S4*
