# Supplementary figures and images for: Increased Monocyte-Derived CD11b+ Macrophage Subpopulations Following Cigarette Smoke Exposure Are Associated With Impaired Bleomycin-Induced Tissue Remodelling
Source: Front Immunol. 2021 Sep 16;12:740330. doi: 10.3389/fimmu.2021.740330 (PMC8481926; doi:10.3389/fimmu.2021.740330)

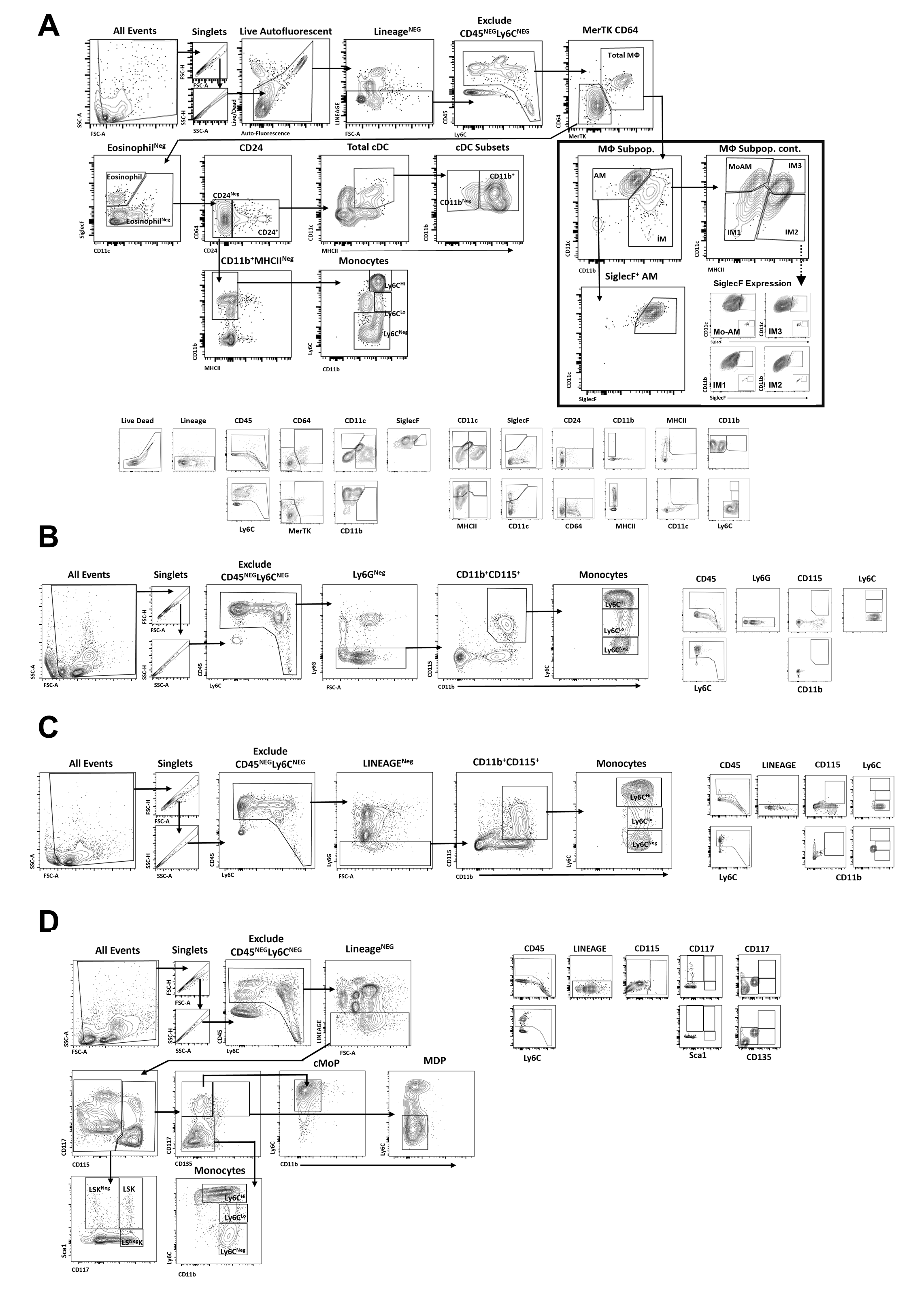

Supplement: Supplementary Figure 1 — Monocyte and macrophage subpopulations gating strategies. (A) Lungs were enzymatically digested and stained for flow cytometry. Diagrams showing gating strategies used to isolate total macrophage populations identified as Live autofluorescent (FITC) +/-LineageNegCD45+MertK+CD64+. Macrophage lineage includes CD3, CD19, NK1.1, EpCAM and Ly6G. We further distinguished CD11c+CD11bNegSiglecFHi resident alveolar macrophages (Res-AM), CD11c+CD11b+MHCIINeg monocyte-derived alveolar macrophages (Mo-AM), CD11cNegCD11b+MHCIINeg interstitial macrophage 1 (IM1), CD11cNegCD11b+MHCII+ (IM2), CD11c+CD11b+MHCII+ (IM3) plus Ly6CLo, Ly6CHi monocyte subsets. Blood (B) and spleen (C) monocyte populations were determined as CD45+Ly6GNegCD11b+CD115+ then Ly6CLo and Ly6CHi. (D) Bone marrow monocytes and progenitors were defined as CD45+LineageNeg. Subsequently monocytes defined as CD11b+CD115+CD117NegCD135Neg then Ly6CLo and Ly6CHi. Bone marrow lineage includes CD3, CD19, NK1.1, CD90.2 and Ly6G. Myeloid progenitors were defined as CD115+CD117+CD135NegCD11bNegLy6CHi common monocyte progenitor (cMoP), CD115+CD117+CD135+CD11bNegLy6CNeg monocyte-macrophage dendritic cell progenitor (MDP) plus CD115NegCD117+Sca1Hi Sca1 c-Kit (LSK), CD115NegCD117+Sca1Neg LSNegK and CD115NegCD117NegSca1+ LSKNeg. Fluorescence minus one was used to gate each of the population of interest. [file Image_1.tif]

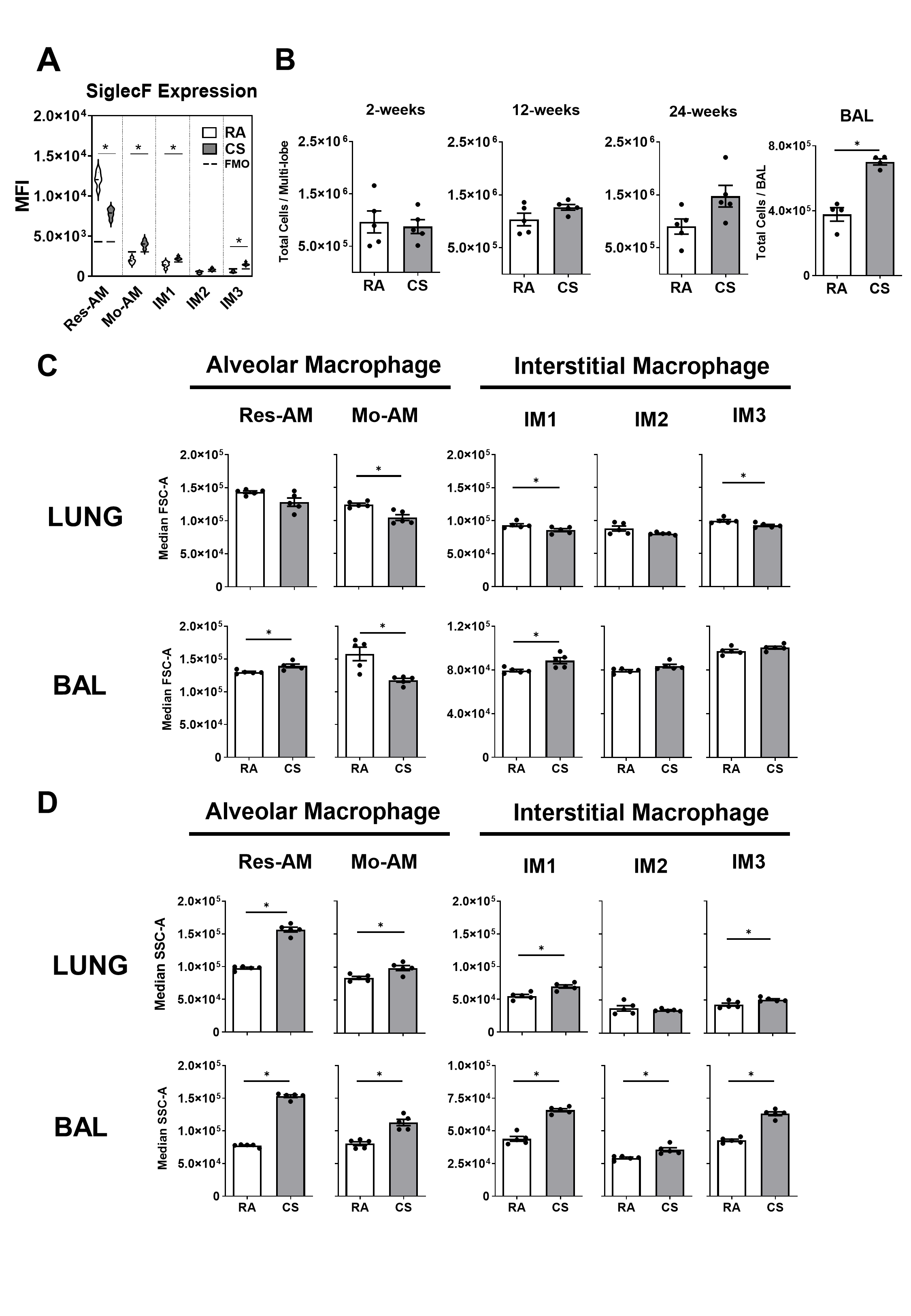

Supplement: Supplementary Figure 2 — Macrophage size and granularity following cigarette smoke exposure. (A) Median fluorescent intensity of siglecF expression for all macrophage subpopulations at 12 weeks CS exposure. Data show total numbers of (B) lung (2-, 12-, 24-weeks) and BAL (8-weeks) cells following CS exposure. (C) Res-AM, Mo-AM, IM1, IM2, and IM3 population size measured by forward scatter (FSC). (D) Res-AM, Mo-AM, IM1, IM2, and IM3 population granularity measured by side scatter (SSC). Data show mean ± SEM, n = 5. Unpaired t test with Welch’s correction. MFI, median fluorescent intensity; RA, room air; CS, cigarette smoke. [file Image_2.tif]

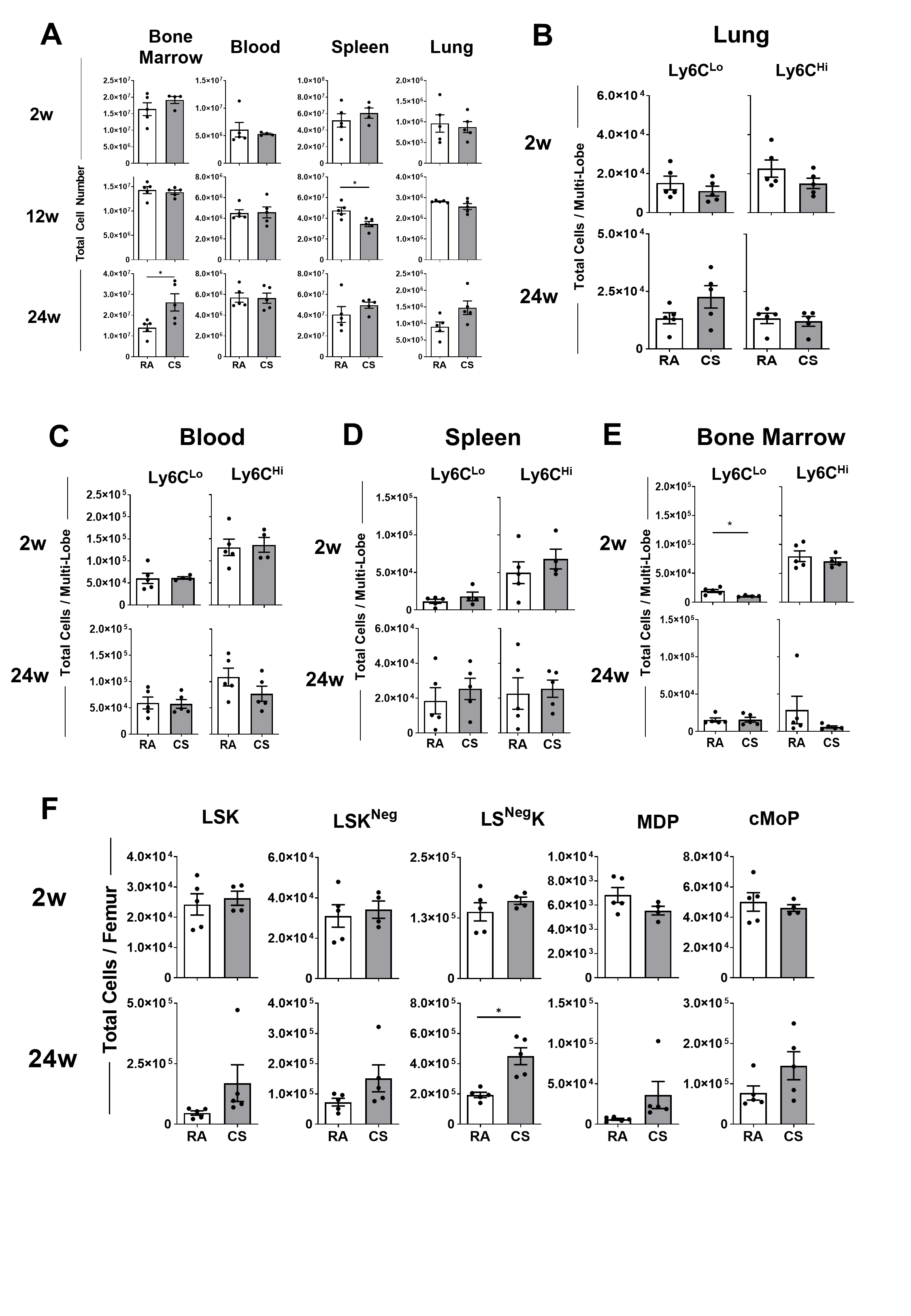

Supplement: Supplementary Figure 3 — Progenitor and monocyte populations at 2, 12 and 24-weeks following cigarette smoke exposure. Female C57BL/6 mice were RA or CS-exposed for 2, 12, or 24-weeks. Data show total cell numbers in (A) lung, blood, spleen, and bone marrow. Data also show total numbers of Ly6CLo and Ly6CHi monocyte populations in (B) lung, (C) blood, (D) spleen, and (E) bone marrow and (F) total numbers of macrophage progenitor cells in the bone marrow (lineage-negative Sca1 c-Kit (LSK), monocyte-macrophage dendritic cell progenitor (MDP), common monocyte progenitor (cMoP)). Data show mean ± SEM, n= 4 - 5. Unpaired t test with Welch’s correction. RA, room air; CS, cigarette smoke. [file Image_3.tif]

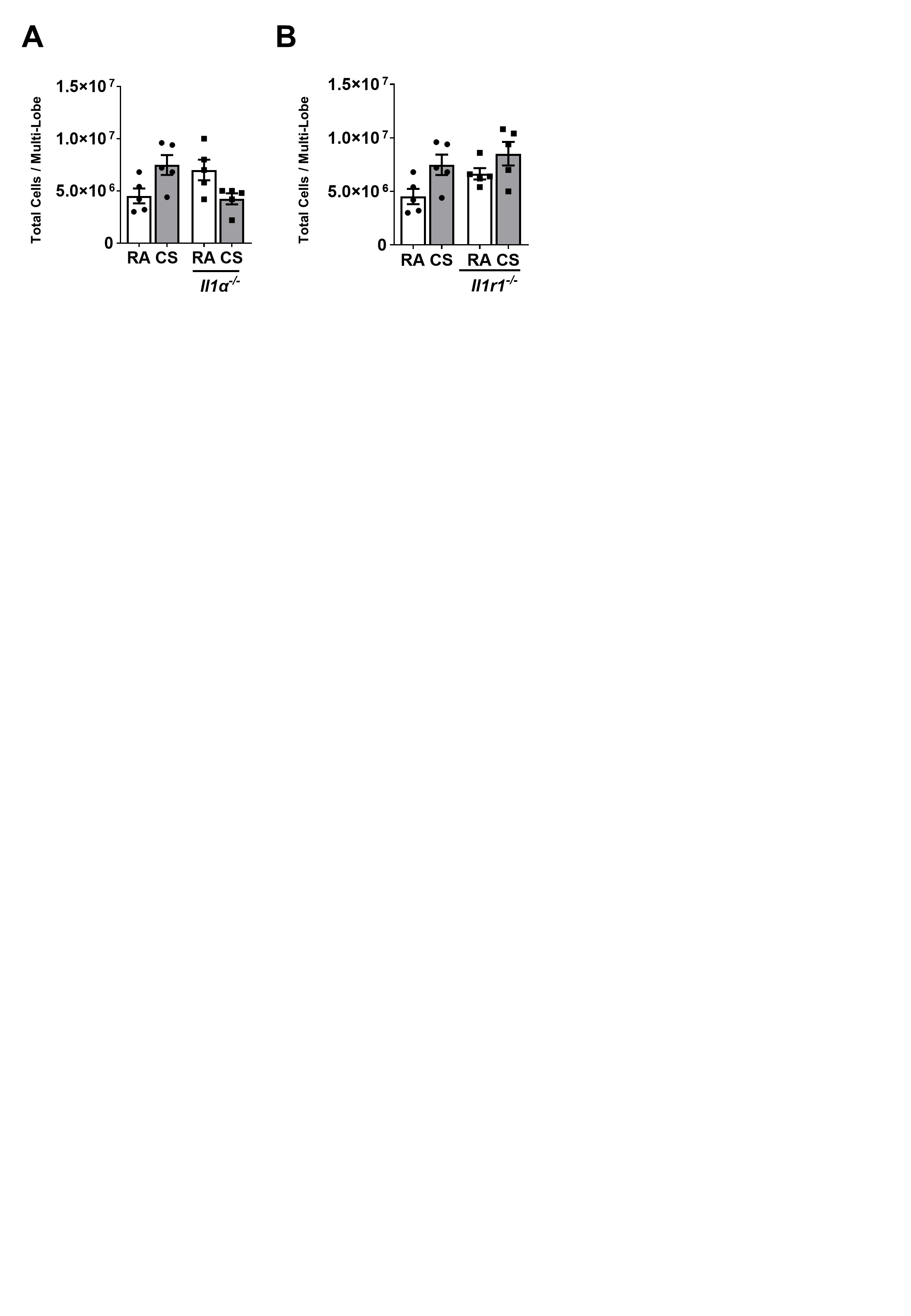

Supplement: Supplementary Figure 4 — Pulmonary macrophages are expanded via IL1α during cigarette smoke exposure. Female C57BL/6, Il1a-/- and Il1r1-/- mice were RA or CS-exposed for 8-weeks. (A) Data show total cell number in (A) Il1a-/- and (B) Il1r1-/- mice plus C57BL6 wildtype controls. Data show mean ± SEM, n = 5. Two-way ANOVA with Tukey’s multi-comparison test. RA, room air; CS, cigarette smoke. [file Image_4.tif]

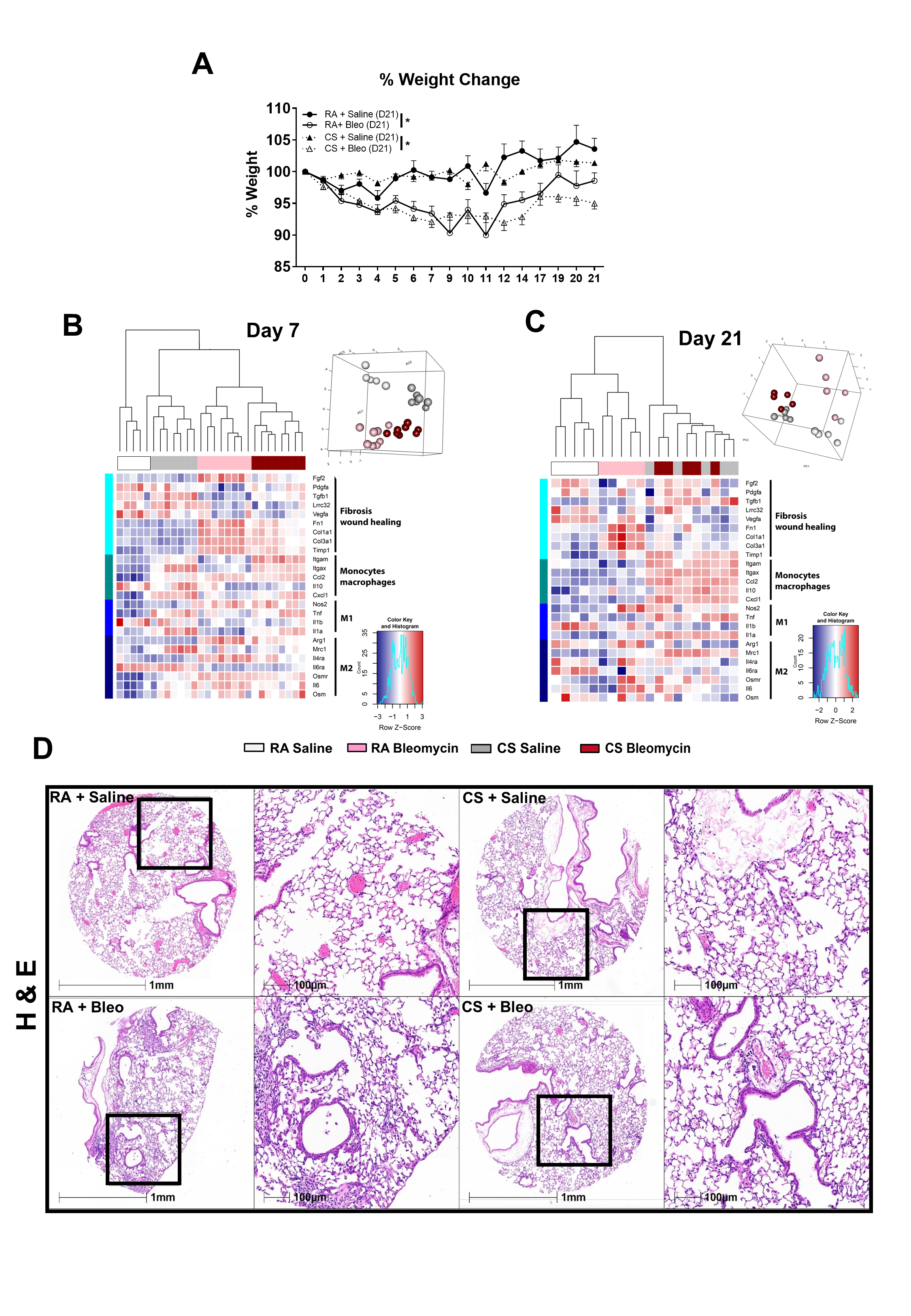

Supplement: Supplementary Figure 5 — Fibrotic measurements at day 21 of bleomycin instillation. C57BL/6 female mice were administered bleomycin (0.05U/mouse) or control saline following 12-weeks of RA or CS exposure. Mice were monitored 21 days prior to flexiVent® lung measurements and tissue harvest. (A) Body weight was measured daily and shown as percentage change in body weight. Principal component analysis and heatmaps defined by 25 mouse genes from lung homogenate assessed by NanoString at (B) day 7 and (C) day 21. (D) Representative images for H&E-stained sections. Data show mean ± SEM, n= 4-10. Two-way ANOVA with Tukey’s multi-comparison test. RA, room air; CS, cigarette smoke. [file Image_5.tif]

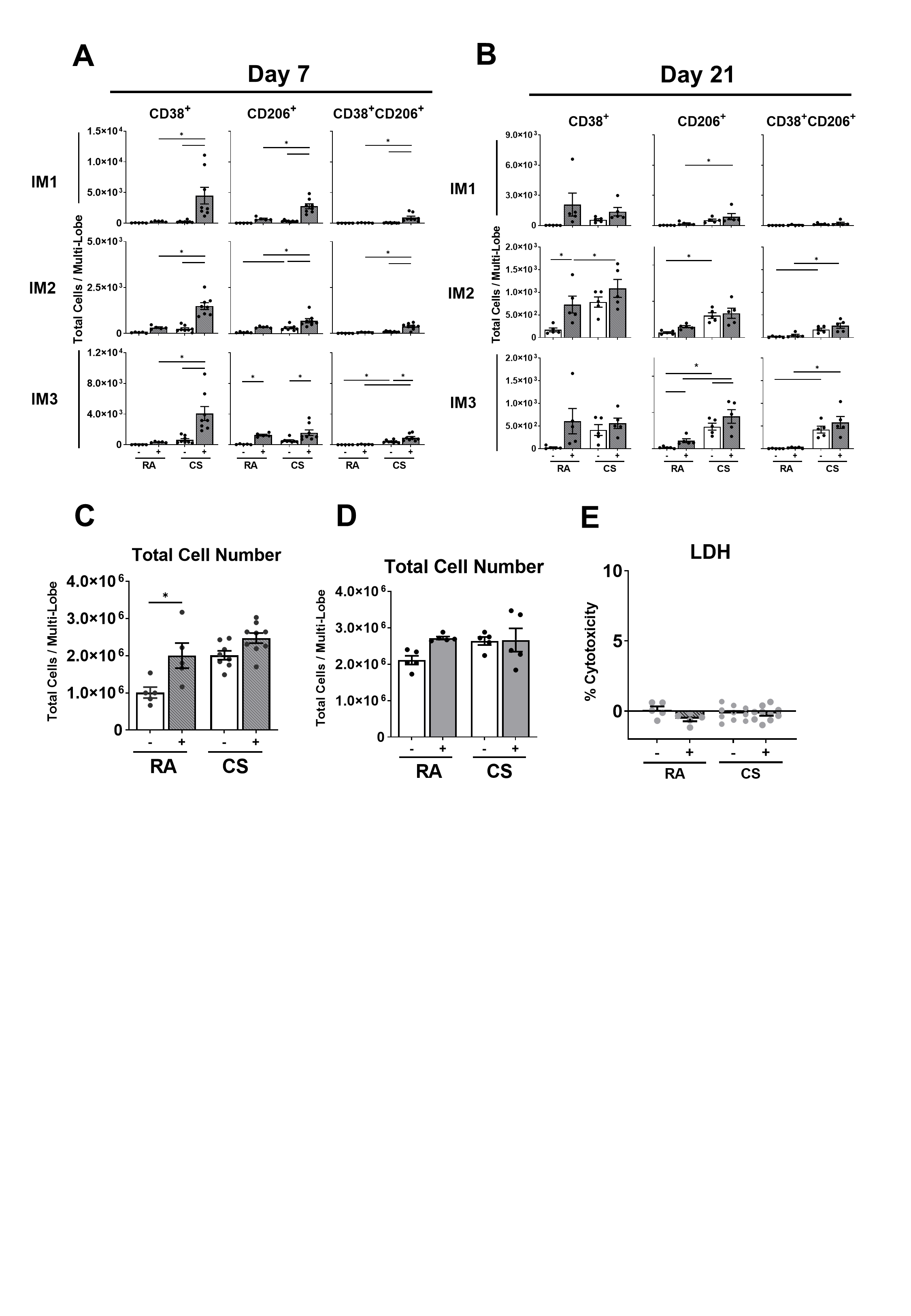

Supplement: Supplementary Figure 6 — CD38+ macrophages are increased in cigarette smoke-exposed bleomycin-treated mice. C57BL/6 female mice were administered bleomycin (0.05U/mouse) or control saline following 12-weeks of RA or CS exposure. Graphs demonstrate total numbers of IM1, IM2, and IM3 populations expressing CD38, CD206 and CD38/CD206 in lung tissue following (A) 7 days or (B) 21 days of bleomycin administration. Total lung cell counts (C) day 7 and (D) day 21. Adherent lung CD45+ lactose dehydrogenase release in ex vivo TGF-β1, IL-6 and IL-4 stimulated cell supernatant from (E) 12-week room air (RA)- or cigarette smoke (CS)-exposed mice. Shown mean ± SEM, n = 4 - 10. Two-way ANOVA with Tukey’s multi-comparison test. RA, room air; CS, cigarette smoke. [file Image_6.tif]
